# Emergency thoracotomies in traumatic cardiac arrests following blunt trauma – experiences from a German level I trauma center

**DOI:** 10.1007/s00068-023-02289-7

**Published:** 2023-06-03

**Authors:** Marcel Niemann, Frank Graef, Fabienne Hahn, Elisa Celine Schilling, Tazio Maleitzke, Serafeim Tsitsilonis, Ulrich Stöckle, Sven Märdian

**Affiliations:** 1 Charité–Universitätsmedizin Berlin, Corporate Member of Freie Universität Berlin and Humboldt-Universität Zu Berlin, Center for Musculoskeletal Surgery, Augustenburger Platz 1, 13353 Berlin, Germany; 2 Berlin Institute of Health at Charité–Universitätsmedizin Berlin, Julius Wolff Institute, Augustenburger Platz 1, 13353 Berlin, Germany; 3 Berlin Institute of Health at Charité–Universitätsmedizin Berlin, BIH Biomedical Innovation Academy, BIH Charité Clinician Scientist Program, Anna-Louisa-Karsch-Straße 2, 10178 Berlin, Germany

**Keywords:** Multiple trauma, Thoracotomy, Resuscitative thoracotomy, Traumatic cardiac arrest

## Abstract

**Purpose:**

Resuscitative thoracotomies (RT) are the last resort to reduce mortality in patients suffering severe trauma. In recent years, indications for RT have been extended from penetrating to blunt trauma. However, discussions on efficacy are still ongoing, as data on this rarely performed procedure are often scarce. Therefore, this study analyzed RT approaches, intraoperative findings, and clinical outcome measures following RT in patients with cardiac arrest following blunt trauma.

**Methods:**

All patients admitted to our level I trauma center's emergency room (ER) who underwent RT between 2010 and 2021 were retrospectively analyzed. Retrospective chart reviews were performed for clinical data, laboratory values, injuries observed during RT, and surgical procedures. Additionally, autopsy protocols were assessed to describe injury patterns accurately.

**Results:**

Fifteen patients were included in this study with a median ISS of 57 (IQR 41–75). The 24-h survival rate was 20%, and the total survival rate was 7%. Three approaches were used to expose the thorax: Anterolateral thoracotomy, clamshell thoracotomy, and sternotomy. A wide variety of injuries were detected, which required complex surgical interventions. These included aortic cross-clamping, myocardial suture repairs, and pulmonary lobe resections.

**Conclusion:**

Blunt trauma often results in severe injuries in various body regions. Therefore, potential injuries and corresponding surgical interventions must be known when performing RT. However, the chances of survival following RT in traumatic cardiac arrest cases following blunt trauma are small.

## Introduction

Resuscitative thoracotomies (RT) are an *ultima ratio* in haemodynamically unstable patients or traumatic cardiac arrest. The procedure was first introduced to control intrathoracic hemorrhage following penetrating trauma. It allows quick access to a cardiac tamponade, commonly followed by a pericardiotomy [1, 2]. Today, RTs are also performed in blunt trauma cases with cardiac arrests, with reported survival rates of up to 25.4% [3]. However, most studies found higher mortality rates and worse neurological outcomes when comparing RTs following blunt compared to penetrating trauma [4, 5].

RTs are recommended in patients with cardiac arrest following (a) blunt trauma and less than 10 min of pre-hospital cardiopulmonary resuscitation (CPR) or (b) penetrating torso trauma and less than 15 min of pre-hospital CPR [6, 7]. Discussing pre-hospital RTs has led to a recent update of the European Resuscitation Council (ERC) Guidelines for Resuscitation in 2015 [6, 8–11].

However, data on injury patterns and patient outcomes following RT after blunt trauma is scarce. Therefore, this study aimed to analyze all RTs performed following blunt trauma at a German level I trauma center between 2010 and 2021. Emphasis was put on patients' outcomes, intraoperative findings, and procedures performed during RT. Furthermore, we assessed autopsy protocols of deceased patients to describe patients' entire injury pattern and cause of death.

## Methods

Institutional review board approval was obtained before data acquisition (application number EA4/119/20). The patient cohort was selected through our clinical information system (SAP ERP 6.0 EHP4, SAP AG, Walldorf, Germany). According to the German operation and procedure classification system (OPS), surgical procedures are encoded for each patient and can thus be filtered according to OPS terms. Therefore, we searched for the respective OPS codes translating to thoracotomies between 2010 and 2021. Results were further filtered for patients who had received RT in the emergency room (ER) and had suffered blunt trauma. We excluded all patients who suffered from penetrating injuries to the torso and all hemodynamically stable patients transported to the regular operating room (OR).

In Berlin, the emergency medical services (EMS) are run by the Berlin fire department. Following international guidelines like the resuscitation guidelines of the ERC [6], Berlin EMS provides standard operating procedures (SOP) for pre-hospital emergency management [12]. Vehicles of Berlin EMS are equipped with automatic CPR devices (Corpuls CPR®) and surgical sets to perform pre-hospital RT. Among other things, using automatic CPR devices in persisting cardiac arrest and continuing CPR during transportation is recommended. RT can be considered in a traumatic cardiac arrest within the abovementioned periods after trauma [12]. It must be stated that the surgical sets provided by Berlin EMS only allow pericardiotomy following RT. Resuscitative endovascular balloon occlusion of the aorta (REBOA), aortic cross-clamping, or further surgical interventions are not possible.

In our center, the ER is operational 24/7, with a fully equipped OR embedded within the regular emergency department. Patients with persistent traumatic cardiac arrest are directly transferred to the OR in the ER. The goal in this setting is to establish an organized cardiac rhythm and stable vital signs as fast as possible. To provide standardized equipment for such scenarios, we recently designed the Berlin Acute Trauma Care Instrument Set [13, 14]. This instrument set is mainly used in this specific ER and allows us to perform most surgical interventions. Based on the presumed extent and injury pattern, an RT may be chosen if the patient suffers from cardiac arrest. An anterolateral approach is employed in patients with injuries suspected of being strictly unilateral. In addition, the left anterolateral thoracotomy is regularly used for rapid aortic cross-clamping. Injuries assumed to affect the cardiac box exclusively are approached via sternotomy. The clamshell thoracotomy is chosen when the injury pattern is unlikely to be restricted to one side of the thorax or extensive injury patterns. These are usually expected to be unaccessible via a unilateral approach. However, the surgeons performing the thoracotomy may choose their preferred approach based on the individual case. All RTs are performed by attending surgeons regularly trained in internationally established courses. These include basic skills in Advanced Trauma Life Support (ATLS) and specific surgical skills of the Definitive Surgical Trauma Care (DSTC), and the Advanced Surgical Skills for Exposure in Trauma (ASSET) program. Attending surgeons repeatedly participate in these courses to ensure theoretical and practical knowledge and surgical proficiency for RTs.

Patient data were analyzed for specific baseline characteristics, including age, sex, and Glasgow Coma Scale (GCS) at admission. We assessed vital signs, blood gases, and blood tests at ER admission and discharge. Vital signs included systolic blood pressure [mmHg] and heart rate [/min]. Blood gases comprised hemoglobin (Hb) [g/dl], lactate concentration [mg/dl], base excess (BE) [mmol/l], and pH. Blood tests focused on the International normalized ratio (INR). Last, we counted the number of administered blood products (packed red blood cells (pRBC), fresh frozen plasma (FFP), and platelet concentrates (PC)) in the ER. Surgical protocols were reviewed, and all procedures performed during RT were documented. To evaluate patients' outcomes, we assessed the duration of survival and the neurological outcome of all patients who survived using the Glasgow Outcome Scale (GOS). The GOS ranges from 1 to 5 (1 = dead, 2 = persistent vegetative state, 3 = major handicap, 4 = minor handicap, 5 = well recovered).

Each patient’s diagnoses were obtained from medical reports in the form of codes of the 10^th^ revision of the German Modification of the International Classification of Diseases (ICD-10-GM) and radiology reports. All diagnoses were manually converted to the Abbreviated Injury Scale (AIS), version of 2005, published for the TraumaRegister DGU® [15]. Afterward, the Injury Severity Score (ISS) was calculated accordingly [16]. Furthermore, the Trauma Injury Severity Score (TRISS, estimates the probability of survival for blunt or penetrating injuries) [17], the Revised Trauma Score (RTS, quantifies trauma severity based on the GCS, blood pressure, and respiratory rate) [18, 19], and the Revised Injury Severity Classification (RISC, estimates the probability of survival based on demographics, injury severity, vital signs, the performance of CPR, and laboratory measures), version II [20], were calculated. Finally, we assessed all available autopsy protocols for deceased patients. Protocols were screened for injuries and causes of death.

Statistical analysis was performed using GraphPad Prism (GraphPad Prism for macOS, Version 9.3.1, GraphPad Holdings, LLC, San Diego, CA, United States of America). Data distribution was analyzed using histograms, QQ plots, and skewness. Independent samples were assessed using the Mann–Whitney *U* test, dependent samples using the Wilcoxon signed-rank test, and categorical samples using Fisher's exact test. Without weighting, a multiple linear regression model including intercept and main effects of blood gases at hospital admission on the survival duration of patients was applied. Values are presented as median (interquartile range [IQR]) and frequencies as numbers (portion of the whole [%]). All *p*-values are two-tailed, and *p*-values ≤ 0.05 were considered statistically significant.

## Results

The initial search revealed 47 results. Five patients underwent elective thoracotomy for hemothorax treatment several days after the initial trauma. Fifteen patients suffered penetrating injuries to the thorax, of which seven (46.67%) needed RT in the ER, and eight (53.33%) were hemodynamically stable and transported to the regular OR. Five of the remaining 27 patients (18.52%) suffered from blunt trauma but were hemodynamically stable and transported to the regular OR. Last, seven (25.93%) patients from the remaining were excluded from further analysis as the pre-hospital no-flow period was presumably prolonged (e.g., cardiac arrest not noticed) or RT was terminated due to interdisciplinary consensus in the ER.

Finally, RT was performed in 15 patients (male:female 12:3) with cardiac arrest following blunt trauma. While six patients (40%) fell from great heights, nine (60%) were in road accidents. Of these, five motorcyclists (33%) and three bicyclists (20%) had collisions with cars, respectively, and one pedestrian was run over by a truck (7%). The cohort's median age was 43 years (IQR 25–64), and the primarily documented median GCS at admission was 3 (IQR 3–3). The first included patient who received RT due to cardiac arrest following blunt trauma was recorded in 2015.

The study cohort's median survival duration was 1.19 h (IQR 0.43–11.3). While three patients survived the first 24 h after trauma, only one survived long-term. The 24-h survival rate was 20%, and one patient survived until discharge (7%). At hospital discharge, the median GOS of the surviving patient was 3 (major handicap). Table 1 compares patients who survived less than 24 h to those who survived at least 24 h.

**Table 1:** Baseline characteristics of the entire study population subdivided by the survival time

		Survival < 24 h (*n* = 12)	Survival ≥ 24 h (*n* = 3)	Statistics
Age [years]		44.5 (IQR 22.25–66.5)	40 (IQR 19–52)	*p* = 0.54
GCS		3 (IQR 3–3)	3 (IQR 3–3)	*p* = 1
Gender	Female (%)	3 (25%)	0 (0%)	*p* = 1
	Male (%)	9 (75%)	3 (100%)	
Systolic blood pressure [mmHg]	at ER admission	0 (IQR 0–0)	80 (IQR 50–110)	*p* = 0.05
	at ER discharge	0 (IQR 0–105)	125 (IQR 100–150)	*p* = 0.1
Heart rate [/min]	at ER admission	0 (IQR 0–0)	125 (IQR 120–130)	*p* = 0.02
	at ER discharge	0 (IQR 0–105)	105 (IQR 80–130)	*p* = 0.11
Haemoglobin [g/dl]	at ER admission	9.85 (IQR 6.05–12.15)	9.8 (IQR 4.6–10.1)	*p* = 0.71
	at ER discharge	8.2 (IQR 7.63–9.33)	8.8 (IQR 7.6–13.1)	*p* = 0.81
Lactate [mg/dl]	at ER admission	121.5 (IQR 99.25–136.5)	87 (IQR 22–104)	*p* = 0.07
	at ER discharge	130 (IQR 93–173)	106 (IQR 90–122)	*p* = 0.64
Base excess [mmol/l]	at ER admission	– 24.05 (IQR to 26.75 to – 16.23)	– 14.5 (IQR – 21.1 to – 9.9)	*p* = 0.1
	at ER discharge	– 17.65 (IQR – 20.38 to – 13.45)	– 12 (IQR – 15.7 to 2.1)	*p* = 0.1
pH	at ER admission	6.7 (IQR 6.63–6.8)	6.97 (IQR 6.8–7)	*p* = 0.07
	at ER discharge	7.02 (IQR 6.78–7.1)	7.38 (IQR 6.8–7.4)	*p* = 0.31
INR	at ER admission	3.51 (IQR 1.43–6.08)	1.7 (IQR 1.06–8)	*p* = 1
	at ER discharge	1.58 (IQR 1.57–1.58)	2.08 (IQR 1.78–2.37)	*p* = 0.33
PRBC transfusion [n]		8 (IQR 5–12)	14 (IQR 8–20)	*p* = 0.41
FFP transfusion [n]		7 (IQR 3–10)	11.5 (IQR 3–20)	*p* = 0.71
PC transfusion [n]		0 (IQR 0–2)	2 (IQR 0–4)	*p* = 0.64
Highest AIS	Head and neck	4 (IQR 2–5)	3 (IQR 1–5)	*p* = 1
	Face	0 (IQR 0–1)	0.5 (IQR 0–1)	*p* = 1
	Thorax	5 (IQR 4–6)	5 (IQR 4–5)	*p* = 0.67
	Abdomen	3 (IQR 1.5–5)	4 (IQR 3–4)	*p* = 0.64
	Extremity	5 (IQR 3.25–5)	2 (IQR 0–3)	*p* = 0.02
	External	0 (IQR 0–0)	0.5 (IQR 0–1)	*p* = 0.4
ISS		59 (IQR 41–75)	50 (IQR 38–57)	*p* = 0.2
TRISS [%]		0.97 (IQR 0.12–2.04)	6.78 (IQR 5.52–8.03)	*p* = 0.09
RTS		0 (IQR 0–0)	2.2 (IQR 1.47–2.93)	*p* = 0.08
RISC II		0.012 (IQR 0.003–0.059	0.076 (IQR 0.052–0.886)	*p* = 0.048
Duration of survival [h]		1.12 (IQR 0.34–4.41)	58.42 (IQR 36.95–79.88)	*p* = 0.02

*GCS*  Glascow Come Scale, *INR*  International Normalized Ratio, *PRBC*  Packed red blood cells, *FFP*  Fresh frozen plasma, *PC*  Platelet concentrate, *AIS*  Abbreviated Injury Scale, *ISS*  Injury Severity Score, *TRISS*  Trauma Injury Severity Score, *RTS*  Revised Trauma Scale, *RISC II*  Revised Injury Severity Classification version II, *n. c.*  not computable

The first blood sample of each patient was drawn immediately after ER admission. The study population showed a median Hb concentration of 9.8 g/dl (IQR 5.7–10.2), lactate of 110 mg/dl (IQR 87–132), BE of  – 21.5 mmol/l (IQR  – 26.3 to  – 15), pH of 6.7 (IQR 6.7–6.9), and INR of 2.91 (IQR 1.34–6.31). At admission, the median systolic blood pressure was 0 mmHg (IQR 0–62.5), and the median heart rate was 0/min (IQR 0–112.5). Each patient received a median of 8 pRBCs (IQR 5–13), 7 FFPs (IQR 3–11), and 0 PCs (IQR 0–2) in the ER. Patients who survived until ER discharge had a higher median systolic blood pressure (120 mmHg [IQR 98–153] vs. 75 mmHg [0–113]), lactate concentration (111.5 mg/dl [IQR 84.75–173] vs. 104 mg/dl [IQR 62–118]), BE ( – 13.8 mmol/l [IQR  – 18.6 to  – 12] vs.  – 15 mmol/l [IQR  – 21.1 to  – 9.9]), and pH (7.03 [IQR 6.8–7.38] vs. 6.9 [IQR 6.8–7]) compared to ER admission. Further, median heart rate (110/min [IQR 88–135] vs. 115/min [0–123]), Hb concentration (8.5 g/dl [IQR 7.6–9.4] vs. 9.7 g/dl [IQR 5.7–10]), and INR (1.68 [IQR 1.57–2.22] vs. 6.01 [IQR 1.43–7]) were lower following RT compared to ER admission. None of these changes reached the level of significance.

**Fig. 1:**
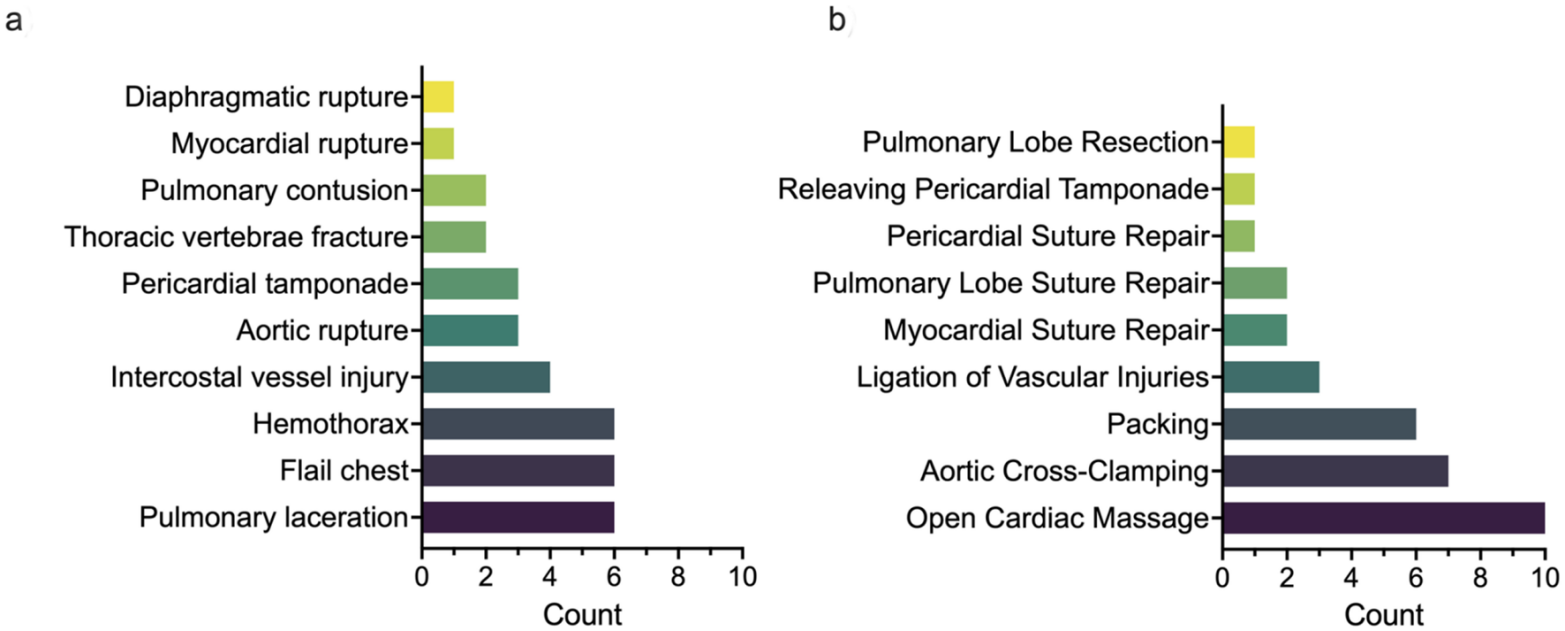
Intraoperative findings and corresponding procedures performed during RT. **a** depicts the intraoperative findings and **b** the surgical procedures. Abbreviations: *RT* Resuscitative thoracotomy

**Fig. 2:**
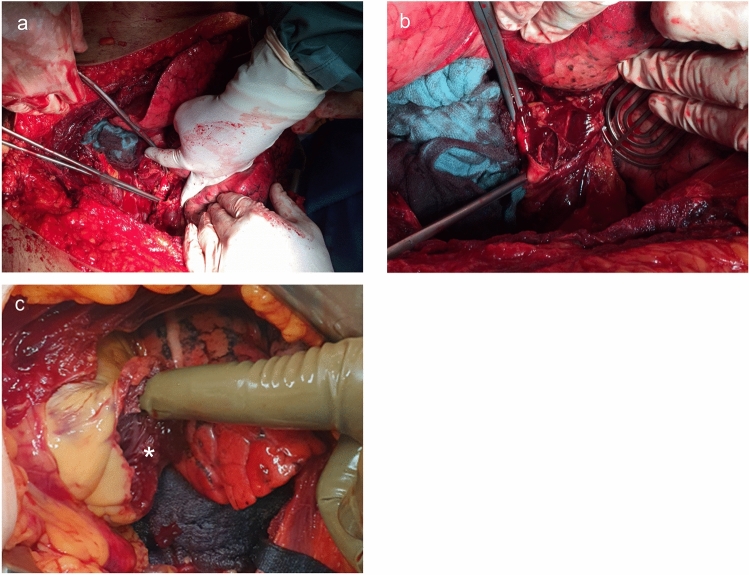
Intraoperative findings after RT. In (**a** and **b)**, a traumatic aorta rupture is displayed, which the patient suffered after falling off a scaffold (approximately 12 m high). In (**c**), a traumatic rupture of the left ventricle is shown, which the patient suffered after being run over by a car. * indicates the specific injuries in each sub-figure

The median highest AIS scores per patient were as follows: Head and neck 4 (IQR 1–5), face 0 (IQR 0–1), thorax 5 (IQR 4–5), abdomen 3 (IQR 3–5), extremity 4 (IQR 3–5), and external 0 (IQR 0–1). The median ISS was 57 (IQR 41–75), TRISS 1% (IQR 0.29–3.78), RTS 0 (IQR 0–2.2), and RISC II 0.02 (IQR 0.003–0.063). In the ER, RT was performed using either an anterolateral (20%), sternotomy (20%) or clamshell approach (60%). The injury patterns observed during RT are displayed in Fig. 1a, and the surgical procedures performed in Fig. 1b. Figure 2 shows representative examples of intraoperative findings during RT.

**Fig. 3:**
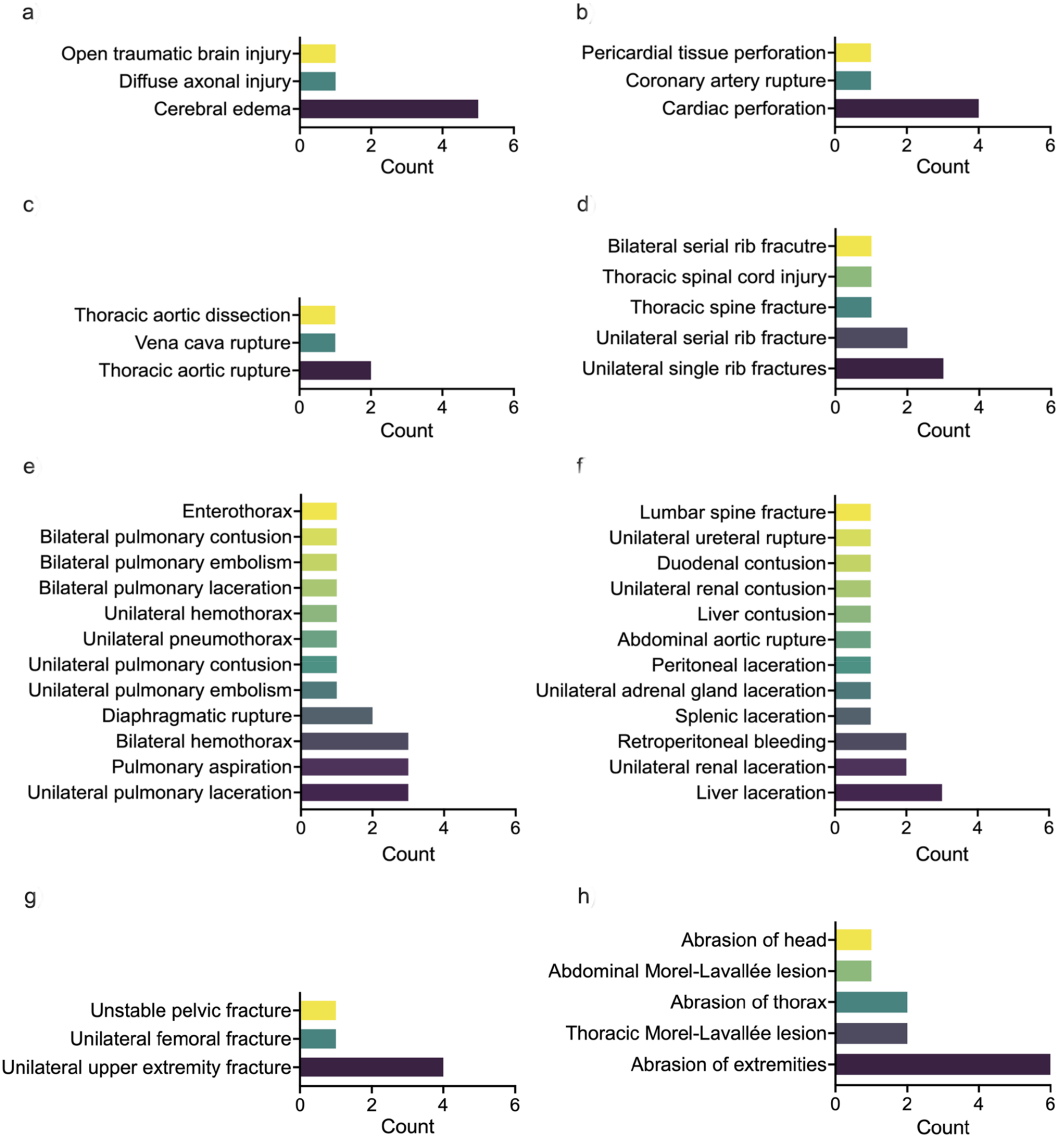
Bar graphs depicting the autopsy findings following thoracotomy for blunt trauma. **a** depicts the count of the head, **b–e** thorax (divided into **b** cardiac, **c** vascular, **d** osseous, and **e** pulmonary injuries of the thorax), **f** abdominal, **g** extremity, and **h** external injuries

We obtained the autopsy protocols for six of the deceased patients. The injury patterns observed during autopsies remarkably differed from the intraoperatively documented injuries. Autopsy protocols described severe injuries at various locations. Especially the thorax and the abdomen were predominantly injured. Further, all but one patient had severe head injuries. Figure 3 displays the total injury patterns documented in the autopsy protocols.

The causes of death after blunt trauma were massive hemorrhage in three patients (50%), central pulmonary embolism in one patient (17%), and open traumatic brain injury in one patient (17%). In the last patient, the cause of death could not be determined (17%).

## Discussion

This study describes patterns and severity of injuries following RT in patients with traumatic cardiac arrest following blunt trauma. Our data show that the survival rates of patients with cardiac arrest due to blunt trauma remain low. Mortality is high even in a big trauma center in an urban environment with short first response and transport times. However, this is the first study to specifically report injury patterns and causes of death from autopsy protocols of deceased patients. These accurately summarize the expected spectrum of injuries when performing RT in blunt trauma patients.

RT in patients who sustained blunt trauma is rarely successful concerning survival. It has been shown that survival rates are lower than in patients with penetrating injuries [4, 21, 22]. Previously published data suggest that RTs could achieve up to 3% survival rates following blunt and up to 9% following penetrating trauma [5]. Lower survival rates after blunt trauma can be explained by the affection of multiple body regions and regularly observed concomitant injuries such as traumatic brain injuries [23]. Our data demonstrate a 24-h survival rate of 20% and an overall survival rate of 7%. Previous TRISS calculations had suggested a median survival rate of 1% for the entire cohort. Recently, Thorsen et al. reported a survival rate of 19% in 20 patients who underwent RT following mainly blunt trauma in a large Norwegian city. In their cohort, TRISS calculation had suggested a survival probability of 8% with a median ISS of 46 [24]. Moreover, Fitzgerald et al. showed that introducing a standardized training program in which trauma surgeons are taught how to conduct RT increased survival rates by up to 25% [25]. Taking the studies mentioned above into account, survival rates of patients with blunt thoracic trauma following RT seem to depend on specific intrahospital settings and the surgeons’ training grade. It needs to be noted, however, that survival probability scores like TRISS calculation regularly overestimate survival rates [26, 27].

We demonstrated that blunt trauma leads to various intrathoracic pathologies. Managing these injuries demands infrastructural requirements and surgical expertise that goes beyond the ones for penetrating injuries [28–30]. Mansour et al. reported aorta and arch vessel tears and pulmonary and cardiac injuries as the most frequently encountered injuries during RT following blunt trauma [31]. Hunt et al. reviewed RTs in blunt thoracic trauma and observed injury patterns that included aortic ruptures, cardiac contusions, tracheobronchial disruptions, ruptured bronchus, and/or cardiac contusions [32]. Both authors' observations are concordant with our data. Therefore, our report underscores the necessity of an experienced interdisciplinary team to manage these complex injury patterns. This team needs to be able to perform organ and vessel repair. Additionally, scrub nurses and ER and OR technicians must be trained in the same techniques. Yet, RT in patients with cardiac arrests following blunt trauma rarely prevents a fatal outcome. Decisions for RT following blunt trauma should therefore be made very restrictively. The same applies to pre-hospital RT in blunt trauma patients, as these rarely die from cardiac tamponade. We reported severe injuries that cannot be sufficiently addressed in a pre-hospital setting. Blunt trauma is Europe’s most commonly observed injury type [33]. Official pre-hospital emergency management guidelines should consider these injuries separately.

We compared the initial laboratory values and hemodynamics of patients surviving at least 24 h and patients surviving less than 24 h after admission. The heart rate at hospital admission was higher in the group surviving at least 24 h. There were no other relevant differences between the groups. Additionally, linear regression did not show a significant influence of initial laboratory parameters on survival duration. Bigger sample sizes are, however, needed in future studies to confirm our findings. In previous studies, witnessing a cardiac arrest [34] and a short duration of resuscitation [34, 35] were commonly observed as positive outcome markers. In non-traumatic cardiac arrest, pH at hospital admission was shown to predict survival [36]. Further, a recently published study by Seewald et al. analyzed traumatic out-of-hospital cardiac arrests in Germany [37]. They found various independent predictors of mortality, including age and sex, ISS, haemodynamic shock in the ER, and the need for resuscitation in the ER.

Concerning the neurological outcome of survivors following RT, previous reports stated an overall good recovery (GOS score of 5) in 68–100% of cases, which mainly included penetrating trauma patients [5, 38]. Due to the high mortality, evidence regarding the neurological outcome after RT for the treatment of blunt trauma is still lacking. Our results demonstrate a moderate recovery (GOS score of 3) at hospital discharge. Thorsen et al. recently demonstrated that four out of five survivors of RT after blunt trauma had a good recovery based on GOS, and one patient showed a moderate disability [24]. Comparability between our study and Thorsen et al. is limited, given the small numbers of survivors in both studies.

The main limitation of this study is the small number of cases and that we did not receive autopsy protocols on all deceased patients, as authorities did not grant access due to ongoing legal cases. Nevertheless, we presented a relatively comprehensive case series of rare injury patterns and their challenging treatment approach. This adds relevant evidence concerning the injury spectrum and potential cause of death following a cardiac arrest after blunt trauma. Last, database analyses of trauma registries such as the German Trauma Registry can provide large-scale information on incidences and prevalences of these injuries [39]. Unfortunately, these data often lack detailed information on intraoperative findings like the ones reported in this study. These limitations need to be considered when interpreting our results.

## Conclusion

This study provided extensive clinical data on patients with blunt thoracic trauma who underwent RT. In addition, we delivered a detailed description of intraoperative findings and surgically performed techniques needed for RT. However, RT in patients who sustained severe blunt trauma shows limited efficacy in preventing mortality. Therefore, RT in patients with cardiac arrest following blunt trauma can be initiated as a last resort during resuscitation, yet a general recommendation cannot be concluded from our data. 

## Data Availability

The data sets used and/or analyzed in the present study are available from the corresponding author upon request.

## Abbreviations

RT: Resuscitative thoracotomy
CPR: Cardiopulmonary resuscitation
ERC: European resuscitation council
OPS: Operation and procedure classification system
ER: Emergency room
GCS: Glasgow coma scale
Hb: Haemoglobin
BE: Base excess
INR: International normalized ratio
pRBC: Packed red blood cells
FFP: Fresh frozen plasma
PC: Platelet concentrate
GOS: Glasgow outcome scale
ICD-10-GM: 10th revision of the German modification of the international classification of diseases
AIS: Abbreviated injury scale
ISS: Injury severity score
TRISS: Trauma injury severity score
RTS: Revised trauma score
RISC: Revised injury severity classification
IQR: Interquartile range
